# Establishing a Pharmacy-Based Patient Registry System: A Pilot Study for Evaluating Pharmacist Intervention for Patients with Long-Term Medication Use

**DOI:** 10.3390/pharmacy6010012

**Published:** 2018-01-25

**Authors:** Manabu Akazawa, Akiko Mikami, Yuri Tamura, Natsuyo Yanagi, Shinichi Yamamura, Hiroyasu Ogata

**Affiliations:** 1 Meiji Pharmaceutical University, Noshio 2-522-1, Kiyose-city, Tokyo 204-8588, Japan; achito@nifty.com (A.M.); d176957@std.my-pharm.ac.jp (Y.T.); n-yanagi@chiba-u.jp (N.Y.); 2 Graduate School of Medical and Pharmaceutical Sciences, Chiba University, Inohana 1-8-1, Chuo-ku, Chiba 260-8670, Japan; 3 Japanese Society for Applied Therapeutics, Hitotsubashi 1-1-1, Chiyoda-ku, Tokyo 100-0003, Japan; yamaroman@gmail.com (S.Y.); hi-ogata@wa2.so-net.ne.jp (H.O.)

**Keywords:** community pharmacy, patient registry, pharmacist intervention, chronic condition, long-term medication

## Abstract

*Background:* In Japan, an increasing number of patients are prescribed a large amount of long-term medications by large hospitals that are then dispensed by a community pharmacy. This practice often leads to considerable wastage of medicine. As part of their professional role, community pharmacists are expected to contribute more to the appropriate use of medication by patients. Using a prospective cohort, we aimed to evaluate pharmacists’ role in the community. *Methods:* We created a patient registry system for community pharmacies to monitor long-term medication use by patients with chronic conditions. Patient drug adherence and potential problems were monitored through regular home visits or telephone calls by the pharmacist at least once a month between patient hospital visits. Patient data were collected and stored in an internet-based system. *Results:* Over a one-year follow-up, 28 out of 37 registered patients from 14 community pharmacies were continuously monitored. In total, we extracted 19 problems relating to medication use, 17 to physical complaints, eight to patient concerns, and two others. *Conclusion:* The registry system was useful for identifying medication-related problems as well as patient concerns and changes in their condition. Pharmacists might play a key role in improving patient care in the community.

## 1. Introduction

The universal health insurance system is a unique feature of the Japanese healthcare system, and it allows patients to access any health services at a relatively low cost [1]. However, this system makes ineffective use of healthcare resources [2]. Many patients with minor disease conditions tend to seek health services at large-scale hospitals instead of local clinics. As a result, patients must wait hours to see doctors for only a minute; this situation is called ‘three-hour wait, three-minute contact’ [3]. In this healthcare environment, patients with chronic conditions often visit the hospital every two or three months and receive prescriptions to fulfil these periods. They then pick up a large amount of medications at a community pharmacy. However, Japan does not have a well-established primary care system that coordinates these patients in receiving long-term health services in the community [4]. Especially, even if a patient’s condition becomes stable, referral from large hospitals to clinics does not function well. We do not have a system for family doctors and it is difficult to find an appropriate doctor in the community to care for one’s condition. In addition, the introduction of a prescription refill system that allows patients to collect their medications repeatedly from a pharmacy without seeing their doctor is still under consideration [5].

Thus, patients with chronic conditions have primary responsibility for managing their medication use. Some patients do not use medications as instructed, while others stop taking them because of concerns about changes in their condition or treatment failures. This often leads to a large amount of wasted medicines, which are not used by patients [6,7,8]. According to a report from the Japan Pharmaceutical Association, the annual cost of the wasted medicines was estimated to be 47.5 billion yen in 2007 [9]. Community pharmacists must play a key role in adherence monitoring for patients requiring long-term medication because these patients cannot consult with doctors until their next appointment. Various studies have suggested that telephone counselling by pharmacists could improve patient adherence as well as their health and economic outcomes [10,11].

In April 2016, a new pharmacy system called ‘Kakaritsuke-yakuzaishi’ (family pharmacist) was introduced in Japan [12]. With this system, patients choose individual pharmacists (which requires an additional charge), who is then given the primary responsibility of managing patients’ medications and health conditions. To be eligible to work as family pharmacists, pharmacists require the following: more than three years of work experience as a community pharmacist, a work time of more than 32 h per week, the requisite training, and involvement in community activities. All of this ensures high-quality services. However, the specific services that family pharmacists should provide to patients are not defined. According to Donabedian’s framework, the quality of professional services can be evaluated in terms of structure, process, and outcome [13]. These requirements for family pharmacists merely ensure the structure of pharmacist services. To ensure high-quality pharmaceutical services, the process (i.e., the types of service/interventions that pharmacists should provide) and the outcomes (i.e., the benefits that patients can expect) should also be defined.

Therefore, to evaluate the pharmacist’s role in community-based care, we established a patient registry system based in community pharmacies to monitor long-term medication use by patients with chronic conditions. This short report describes our experience of the first year of a pharmacist-led intervention using this registry as a pilot study.

## 2. Materials and Methods

We developed a patient registry system based in community pharmacies to evaluate the role of pharmacists in improving medication use among patients with chronic conditions [14]. A total of 14 pharmacists in 14 pharmacies, all of whom are members of the Japanese Society for Applied Therapeutics [15] or the Alliance of Pharmacy Executives [16], voluntarily participated in this pilot study. Patients who had been prescribed medications for 36 days or longer from large-scale hospitals and those who met the inclusion criteria of age 40 years or older, having chronic conditions, and having a potential problem with their medical treatment were selected by these community pharmacists. When patients were registered, information related to their medication adherence, including medication history, health condition, lifestyle, and potential problems/concerns, was obtained through a survey (as an initial assessment). Then, between patients’ hospital visits, pharmacists contacted the patients by telephone calls or home visit at least once a month to check their medication use, as well as physical and mental problems (as follow-up assessments). As described in detail at Appendix A, all information taken from the patients was recorded and shared among the pharmacists via an internet-based system (DropBox^R^). Pharmacists recorded patient information using Excel sheets at initial and follow-up assessments. Since information could be shared among pharmacists in different pharmacies, they could check each other’s records to identify the best approach, to identify appropriate patients for registry, and to provide effective interventions. This study was approved by the Institutional Review Board at Meiji Pharmaceutical University (study number 2515), and written consent was obtained from all participants.

The data were collected by pharmacists from patient registration until the end of the monthly follow-up period—that is, about one year for each patient. Patient characteristics including medication adherence, health condition, and lifestyle were assessed via interviews and described. We counted the number of potential problems/concerns identified by the pharmacists and the number of cases improved by the pharmacists’ interventions as a quantitative analysis. In addition, we described good examples of what kinds of interventions were provided and how they worked as a qualitative analysis. In the case that patients requested a withdrawal, the pharmacist recorded the reason for it in detail. When patients did not attend the next visits or did not answer the phone calls (dropouts), the pharmacists continued to try to contact them or other family members until they were found.

## 3. Results

**Figure 1:**
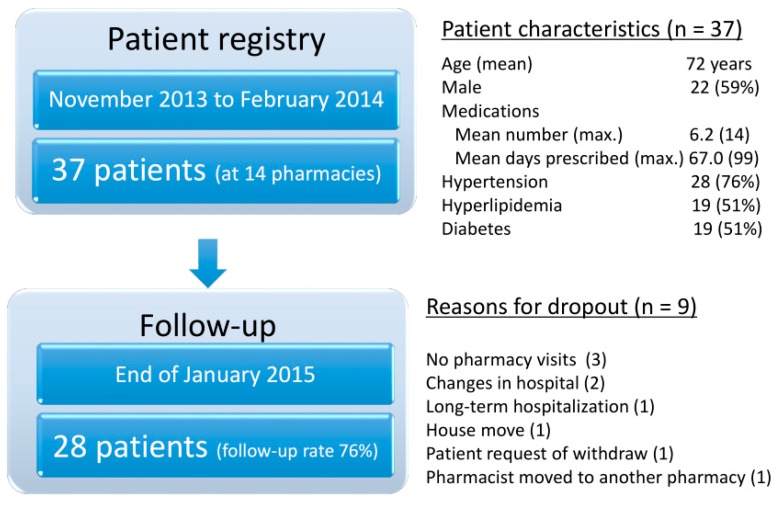
Patient characteristics and participation in the patient registry in the first-year pilot study.

During the four months between November 2013 and February 2014, 37 patients from 14 community pharmacies were registered (mean age = 72 years, 59% male). The mean number of different medications was 6.2 (maximum 14) and the mean duration of prescriptions was 67 days (with a maximum of 99 days). As for the background characteristics of patients, they had at least one chronic condition including hypertension (76%), hyperlipidaemia (51%), or diabetes (51%), as summarized in Figure 1. By the end of January 2015 (the one-year follow-up), 28 patients remained in the registry. The reasons for the nine dropouts were as follows: no patient follow-up data after registration (three cases), changes in hospital (two cases), and one case each of long-term hospitalization, house move, patient request for withdrawal, and the pharmacist moving to another pharmacy.

**Figure 2:**
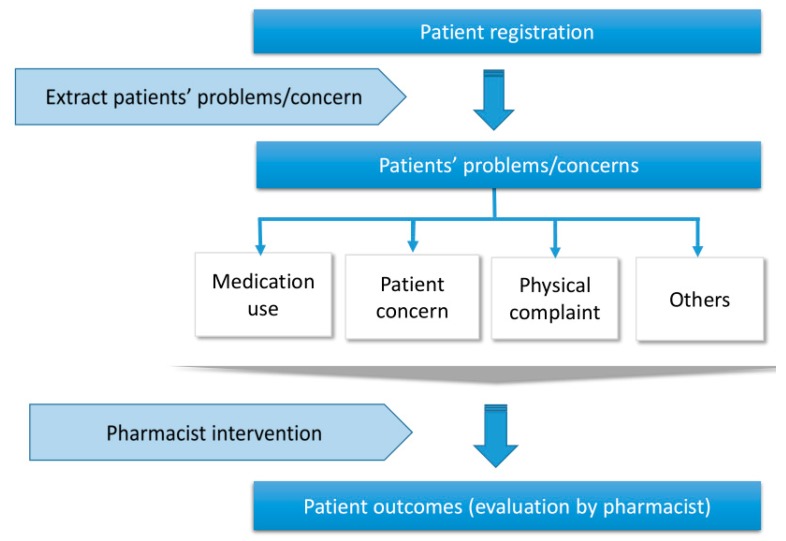
Flowchart of identifying problems and pharmacist interventions.

At the initial assessment, pharmacists suspected problems related to medication uses, patient concerns (e.g., drug safety or effectiveness of medication), physical complaints (e.g., symptom improvement), or others (e.g., lifestyle). In addition, after registration, pharmacists could recognize these problems through close communication with patients during follow-up and provide suggestions or recommendations for improvement as described in Figure 2. In fact, 17 patients were registered due to suspected problems of medications use (non-adherence) and 20 patients were registered by their potential problems (patient concern, physical complaint, others). The potential problems identified by pharmacists during the follow-up period could be classified as medication use, patient concern (eight cases), physical complaints (17 cases), and others (two cases). There were 19 potential medication use problems among 16 patients; 12 cases of forgetfulness, six cases of self-adjustment/interruption, and one case of incorrect medication use. During follow-up, pharmacists discussed with patients as needed, identified why patients could not take medications as instructed, and worked together to look for methods of improvement. In fact, seven problems related to medication use were solved through this approach.

Specific cases are listed as examples of identifying problems, providing pharmacist interventions, and observing patient outcomes in Table 1. Note that we did not use any objective measures to evaluate patient adherence in this pilot study; this was based on the pharmacist’s impression. In addition, lab results were obtained from patients if available. Some clinical results were based on the changes in lab data. However, most of the clinical findings were based on the pharmacists’ judgments based on discussions with patients.

The pharmacists involved in the pilot study were able to identify many potential problems among patients who received a large amount of medications, but could not consult with doctors and pharmacists for a long time. These problems are related to patient health conditions, medication use, and safety concerns. Some patient problems were identified during the follow-up telephone calls. When dispensing medications at the pharmacy, pharmacists did not have enough time to discuss these potential problems or ask about patients’ concerns. However, when pharmacists were able to establish a good relationship with patients through frequent contact, patients gradually began to speak more about their daily life and medication-related problems. In some cases, pharmacists found many unused drugs at the patient’s home, even though the patients had reported good adherence when asked at the pharmacy counter. Notably, in one case, a male patient reported an unusual condition after taking medication; based on his account, the pharmacist suspected that the patient had angina and reported it to his doctor, leading to early diagnosis and treatment.

## 4. Discussion

This pilot study was conducted to establish a pharmacy-based patient registry system, and then, this system was used to evaluate pharmacist interventions to improve potential problems related to long-term medication use. Therefore, we attempted to collect real-world evidence about potential problems of patients, pharmacist interventions, and patient outcomes. We determined what kinds of information should be collected and how often pharmacists should contact patients. In addition, we did not evaluate effectiveness of pharmacist interventions objectively and there were no comparison groups. Thus, it was difficult to determine whether observed outcomes were due to the intervention. However, from this pilot study, we could identify target patient populations and outcome measures that had been used for the new registration system.

One of the merits of this registry system is that pharmacists can learn from each other by sharing information. We asked pharmacists who participated in the pilot study what kinds of information should be collected at the initial and follow-up assessments. In this way, pharmacists could understand how to monitor patients. In addition, the pharmacists needed to collect information about medications prescribed outside of the pharmacies by asking patients or checking patients’ medication notes. Therefore, they had a strong incentive to collect this information to identify patients’ potential problems and monitor them through the registry system.

The patient registry system noted in this study is an effective approach for promoting active intervention by community pharmacists. When pharmacists identify patients who have potential problems and need special care, these patients should be registered and monitored. However, such patient selection and adherence monitoring would create an additional workload for pharmacists. A support system to help pharmacists is essential to continue this registry system. Only a limited number of patients could be recruited and followed-up in the pilot study, which impeded the generalization of the findings. Therefore, we completed the pilot study in March 2015 and developed a new online registry system for initial and follow-up data collection. This new registry system involves nearly 100 pharmacies, and was initiated in May 2015 with 300 registered patients. As of December 2017, the follow-up is still on going.

The goal of the new registry system is to provide a successful model for the family pharmacist from a perspective of quality of professional services, especially the process and the outcomes suggested by Donabedian. Therefore, to evaluate the pharmacists’ roles in community-based care, cumulative evidence is needed showing how many patients have improved relationships with their pharmacists (quantitative data) and how they can establish such good relationships (qualitative data).

## 5. Conclusions

This is the first patient registry system to evaluate the pharmacist’s role in community-based care in Japan. Medication wastage related to long-term prescriptions for chronic conditions is one of the target issues for Japanese healthcare reform, and we expect that family pharmacists might play a key role in promoting appropriate medication use and reducing costs related to wasted medication. Our findings could inform appropriate measures to evaluate health and cost benefits of pharmacist interventions with regard to patient care in the community.

## Figures and Tables

**Table 1:** Examples of potential problems, pharmacist interventions, and patient outcomes.

Category	Potential Problems	Pharmacist Intervention (Suggestion or Recommendation)	Patient Outcomes
**1. Medication Use**			
	Forget to take medicine when eating out	Keep some tablets in the bag constantly	Adherence was improved.
	Forget to take medicine when busy with work	Put the medicine in a conspicuous place Notice that the medicine could be taken also before eating	Adherence was improved.
**2. Concerns**			
	High blood sugar level despite efforts	Wait for the result of the next health check-up, and consider the possibility of hyperglycemia after a meal, as the current average blood sugar level is still better than before	The blood sugar level fell to the normal range on the next measurement.
	Blood pressure variation (low in the morning and high in the night)	Receive counseling from the family doctor	Concern disappeared after hearing that it was not necessary to mind this.
**3. Physical Complaint**			
	Chest ache after exercise	Get medical consultation for angina pectoris fear	The patient underwent detailed examination and was diagnosed and operated on for angina pectoris.
	Dizziness	Drink more water or tea because of possible side effect	Dizziness disappeared after several weeks.
**4. Others**			
	No interest in the results of the health check	Promote health education	The patient became interested in the value of health check-ups (e.g., purchased books); motivation to receive medical treatment increased.

## Appendix A. Data Collection at the Initial and Follow-Up Assessments

At the initial assessment, pharmacists should collect and record the following data.

1. Patient’s ID (automatically assigned at registration)
2. Age and sex
3. Clinical conditions for long-term medication use
4. Date of informed consent
5. Contact information if needed
6. List of all medications used by the patient (not only dispensed medication at the pharmacy, but also those dispensed at different pharmacies)
7. Treatment history
8. Other conditions not for long-term medication use
9. Lab results (blood pressure, lipid, HbA1c, etc.)
10. Treatment conditions (one-pack doses, number of clinics used, etc.)
11. Living environments related to treatment (living alone, job, home care, etc.)
12. Potential problems reported by patient
13. Potential concerns identified by pharmacist
14. Preferred methods for follow-up contacts
15. Others

At the follow-up assessments, pharmacists should monitor and record the following data.

1. Date of contacts
2. Method for monitoring (telephone or visit)
3. Changes in medications
4. Patient reported adherence or number of medications not used
5. Reason for not taking medications as instructed
6. Conditional changes (if any)
7. Lab results (reported by patients)
8. Pharmacist advice given to patients
9. Next appointment dates

When patients request a withdrawal, or have no further pharmacy visits, pharmacists should ask the reasons at the last visit or try to contact patients or family members as much as possible.
